# Effect of Different Bone Graft Materials on Buccal Wall Dimensions During Socket Preservation: A 3D Retrospective Pilot Study

**DOI:** 10.7759/cureus.99927

**Published:** 2025-12-23

**Authors:** Moustapha Jaber, Robin el Jalkh, Carla Maria Khairallah, Adam Saleh, Georges Bou Jaoude

**Affiliations:** 1 Oral Surgery, Saint Joseph University of Beirut, Beirut, LBN; 2 Digital Dentistry, AI and Evolving Technologies, Saint Joseph University of Beirut, Beirut, LBN; 3 Periodontology, Saint Joseph University of Beirut, Beirut, LBN

**Keywords:** alveolar bone remodeling, alveolar ridge resorption, bone grafting substitutes, buccal wall dimensions, socket preservation, superimposition techniques

## Abstract

Introduction

Socket preservation is a fundamental procedure in dental implantology aimed at minimizing post-extraction alveolar ridge resorption. Buccal wall integrity plays a crucial role in implant stability and esthetic outcomes. However, limited research has compared the effects of different bone graft materials on buccal wall preservation. The objective of this study is to evaluate and compare the impact of three bone graft materials, namely autografts, allografts, and xenografts, on buccal wall dimensions in socket preservation procedures using cone beam computed tomography (CBCT) and AI-based superimposition techniques.

Methodology

A retrospective comparative cohort study was conducted on 23 patients who underwent socket preservation between 2017 and 2024. Patients were categorized into three groups based on graft material: allografts, xenografts, and autografts. High-resolution preoperative (T0) and six months postoperative (T1) CBCT scans were analyzed using 3D Slicer® software. Superimposition techniques enabled precise measurement of buccal wall thickness at three levels (L2, L4, L6). Statistical analyses, including Kruskal-Wallis and Wilcoxon signed-rank tests, were performed to assess differences in buccal wall dimensions and resorption rates.

Results

At follow-up (T1), xenografts exhibited significantly better preservation of buccal wall thickness at L2 compared to autografts and allografts (*P *= 0.02 and *P *= 0.04, respectively). However, no significant differences were observed at deeper levels (L4, L6) (*P *> 0.05). Buccal wall resorption occurred in all groups, but no statistically significant differences in overall resorption rates were found (*P *> 0.05). Buccal wall dimensions significantly decreased from T0 to T1 at all levels (L2, L4, and L6) (*P* < 0.001).

The findings suggest that xenografts offer superior preservation at the coronal level, making them a preferred choice in esthetic areas. However, all graft materials showed comparable effects in deeper regions over time. The absence of significant differences in overall resorption rates may be attributed to the study’s small sample size and follow-up duration. These results align with prior literature indicating the prolonged structural support of xenografts but also highlight the ongoing challenge of post-extraction bone loss.

Conclusions

This study underscores the importance of graft material selection in socket preservation, particularly for maintaining coronal ridge dimensions. Despite grafting, significant buccal wall resorption was observed, emphasizing the potential need for additional augmentation techniques. Future research with larger sample sizes and longer follow-up periods is essential to refine clinical protocols and improve long-term implant success.

## Introduction

Socket preservation is a key procedure in dentistry aimed at maintaining alveolar ridge dimensions after extraction [1]. Bone resorption begins post-extraction immediately, compromising implant placement and ridge esthetics [2]. About half of the ridge width is lost within the first year, with 50% of this loss occurring in the first three months [3]. These dimensional changes often necessitate additional surgeries such as bone grafting [3]. Socket preservation, or alveolar ridge preservation, involves filling the extraction socket with graft materials to reduce these changes and support ridge structure [3], offering clear functional and esthetic benefits for prosthetic rehabilitation [3].

The success of this approach depends not only on graft selection but also on the extraction site anatomy [4]. The cribriform plate, a dense lamellar bone anchoring Sharpey’s fibers, is essential for force distribution and vascular supply [4,5]. Its rapid resorption contributes to ridge remodeling and potential buccal wall collapse [5,6]. The buccal wall plays a pivotal role in implant stability, occlusal load distribution, and ridge integrity [6]. A minimum thickness of 2.0 mm is recommended to limit resorption, though often difficult due to thin biotypes or prior bone loss [7,8]. Adequate buccal dimensions enhance peri-implant health, reduce complications such as peri-implantitis, and correlate with higher implant survival and esthetic outcomes [8].

To counter bone loss, various grafting materials are used: allografts, xenografts, autografts, and alloplasts [9]. Allografts, from human donors, provide biocompatibility and moderate resorption with minimal rejection risks [10]. They exist as freeze-dried bone allografts (FDBA), mainly osteoconductive, and demineralized freeze-dried allografts (DFDBA), exposing BMPs for osteoinduction but with less structural strength [11]. Xenografts, mainly bovine, offer a durable scaffold and high osteoconductivity but remodel slowly and may raise safety concerns [10,12,13]. Autografts, harvested from intraoral sites, remain the gold standard due to their osteogenic, osteoinductive, and osteoconductive properties [12,14], though limited by morbidity and infection risks [14]. Alloplasts, synthetic materials, provide customizable resorption and biocompatibility, though integration varies with composition [12].

Advances in imaging, especially cone beam computed tomography (CBCT), enable precise, objective assessment of bone morphology [15,16]. CBCT surpasses 2D imaging in evaluating bone resorption, graft integration, and ridge dimensions [15,16]. Superimposed CBCT scans in DICOM format are indispensable in orthodontic and craniofacial follow-ups [17,18]. Using Voxel-based registration, a technique that aligns volumetric datasets by analyzing voxel intensity values, clinicians can accurately track skeletal modifications over time, quantifying bone volume, shape, and structural integrity [18]. Tools like 3D Slicer® facilitate point-based registration, measurements, and advanced analyses [19]. AI-driven platforms further enhance segmentation, visualization, and longitudinal monitoring, while also supporting education and multicenter data consistency [19]. Such methods align with initiatives like the Quantitative Imaging Network, ensuring reliability and reproducibility of clinical data [19].

Despite extensive research on socket preservation and bone graft materials, the comparative effects of different graft types on buccal wall dimensions remain insufficiently explored [20,21]. Given the buccal wall's crucial role in esthetic and functional success, understanding these differences is essential. Therefore, this study aims to evaluate the impact of allografts, xenografts, and autografts on buccal wall dimensions in socket preservation procedures and to compare their effectiveness in maintaining buccal thickness and promoting bone regeneration.

The null hypothesis (H0) proposes that different bone graft materials (allograft, xenograft, autograft) do not lead to measurable changes in buccal wall dimensions following socket preservation. Whereas the alternative hypothesis (H1) suggests that different bone graft materials lead to measurable changes in buccal wall dimensions following socket preservation.

## Materials and methods

This pilot retrospective cohort study analyzes socket preservation cases treated between January 2017 and December 2024. It was approved by the Research Ethics Board of Saint Joseph University of Beirut under the approval number Tfemd-2025-37. The retrospective radiographic analysis of the data was conducted between January 2025 and March 2025 at the Craniofacial Research Laboratory (Imaging Unit) in the campus of innovation and physical education at Saint Joseph University, with the collaboration of both the oral and maxillo-facial surgery department and the department of digital dentistry, AI and evolving technologies at the faculty of dental medicine at Saint Joseph University.

Patient cases were divided into three groups based on bone graft material type (allograft, xenograft, autograft) used during socket preservation:

· Group 1 (Allograft): Patients receiving allograft bone substitute.

· Group 2 (Xenograft): Patients receiving xenograft bone substitute.

· Group 3 (Autograft): Patients receiving autograft bone substitute.

Surgical procedure of the collected data

Inclusion Criteria

The inclusion criteria consisted of patients requiring tooth extraction followed by implant placement after 6 months, with available CBCT scans obtained before and after socket preservation (acquired at 5 to 6 months). Eligible patients were non-smokers or light smokers (fewer than 10 cigarettes per day), were over 18 years of age, and had teeth extracted from all sites.

Exclusion Criteria

The exclusion criteria included teeth presenting with acute infections, such as suppuration, severe swelling, or spontaneous bleeding; pregnant or lactating women; patients with medical conditions contraindicating implant surgery; individuals with metabolic bone disorders, including osteoporosis; and patients with severe psychiatric illnesses.

**Figure 1:**
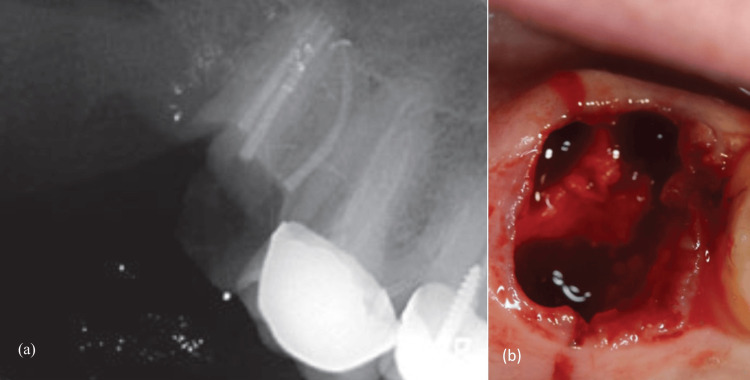
Clinical case example: (a) Pre-treatment radiograph; (b) atraumatic tooth extraction.

On the day of extraction, all patients underwent a preoperative rinse with 0.2% chlorhexidine solution. Local anesthesia was administered using 4% Articaine with 1:100,000 epinephrine. Teeth from all groups were extracted atraumatically using the EX1 piezo insert (Mectron s.p.a., Carasco, Italy) and a syndesmotome to sever the periodontal ligament (PDL) fibers. Teeth were mobilized with elevators and extracted using specialized forceps, without flap elevation or osteotomy. Molars were separated using a Zekrya surgical bur. The socket was curetted to remove residual granulation tissue (Figure 1).

Only sockets presenting a buccal bone thickness between 0.8 and 2 mm at Level L2 were included in this study. This range was chosen to ensure a consistent anatomical baseline that reflects clinically relevant conditions where ridge preservation is most often needed. Sockets with less than 0.8 mm were excluded due to their high risk of complete buccal wall collapse, which could skew the analysis and require additional regenerative procedures beyond simple grafting. Conversely, sites with more than 2 mm of buccal bone were considered less prone to resorption and less dependent on grafting material, potentially biasing the results. By focusing on this intermediate range, the study targeted sockets with moderate vulnerability to resorption, allowing a more accurate evaluation of the true effect of the graft material on buccal wall preservation.

For patients receiving allograft bone, corticocancellous solvent-dehydrated bone allograft (SDBA) (Puros, RTI Biologics, Alachua, FL) with a particle size of 0.25-1.0 mm was rehydrated, packed, and compressed into the socket. A d-PTFE membrane (Cytoplast TXT-200 singles; Osteogenics Biomedical, Lubbock, TX) was applied with minimal tunneling (<3 mm) and secured using the hidden-X suture technique with resorbable poly(glycolide-co-L-lactide) braided sutures (Novosyn, B. Braun, Melsungen, Germany). Primary closure was intentionally omitted.

**Figure 2:**
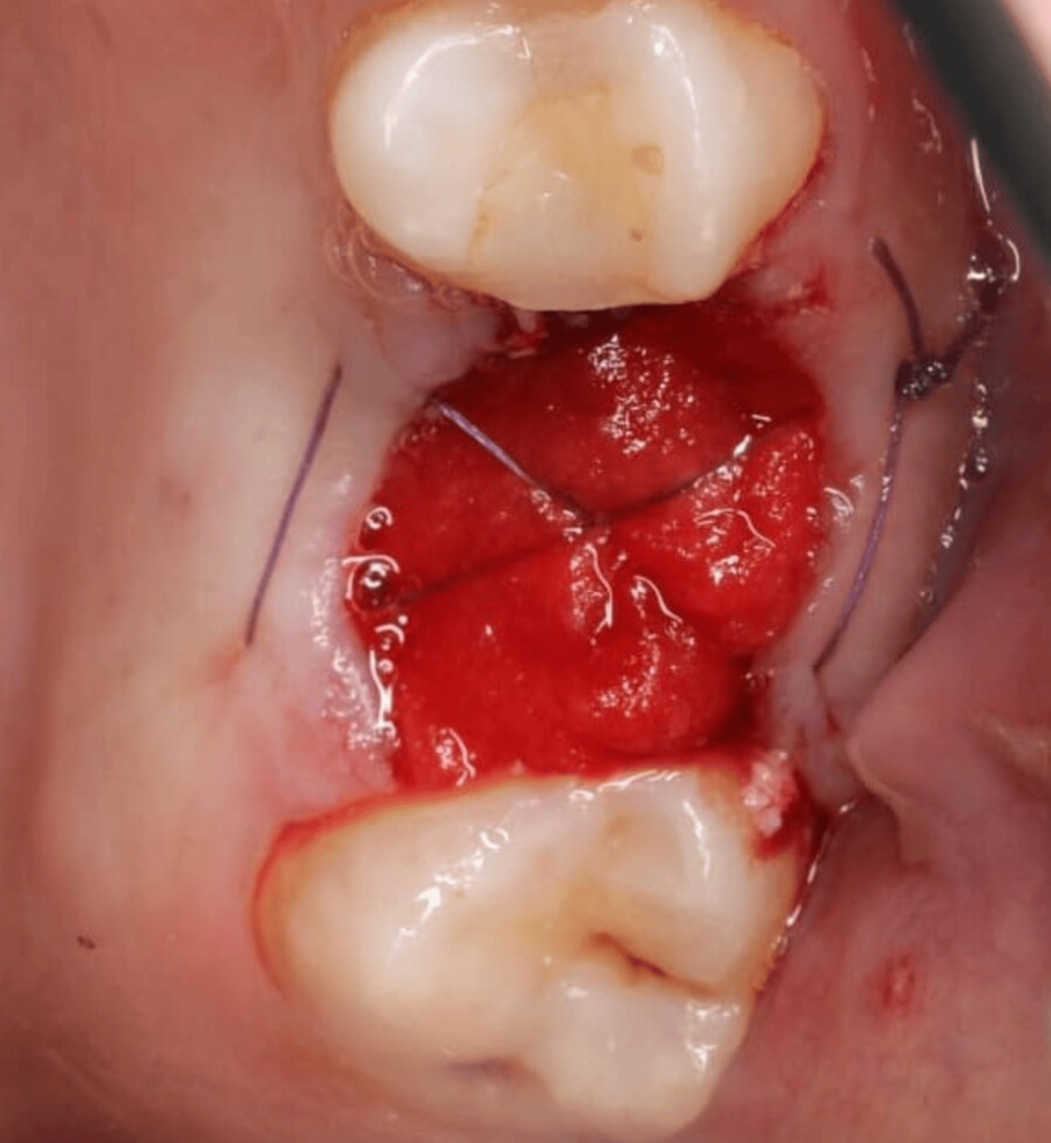
Clinical case example: image illustrating a socket grafted with Bio-Oss xenograft (Geistlich Pharma AG, Wolhusen, Switzerland), covered with a collagen tape and secured using a hidden-X suture.

For xenograft bone, Bio-Oss (Geistlich Pharma AG, Wolhusen, Switzerland) with a particle size of 0.25-1.0 mm was rehydrated, packed, and compressed into the socket. Collagen tape was applied and secured using the hidden-X suture technique with Novosyn sutures (Figure 2).

**Figure 3:**
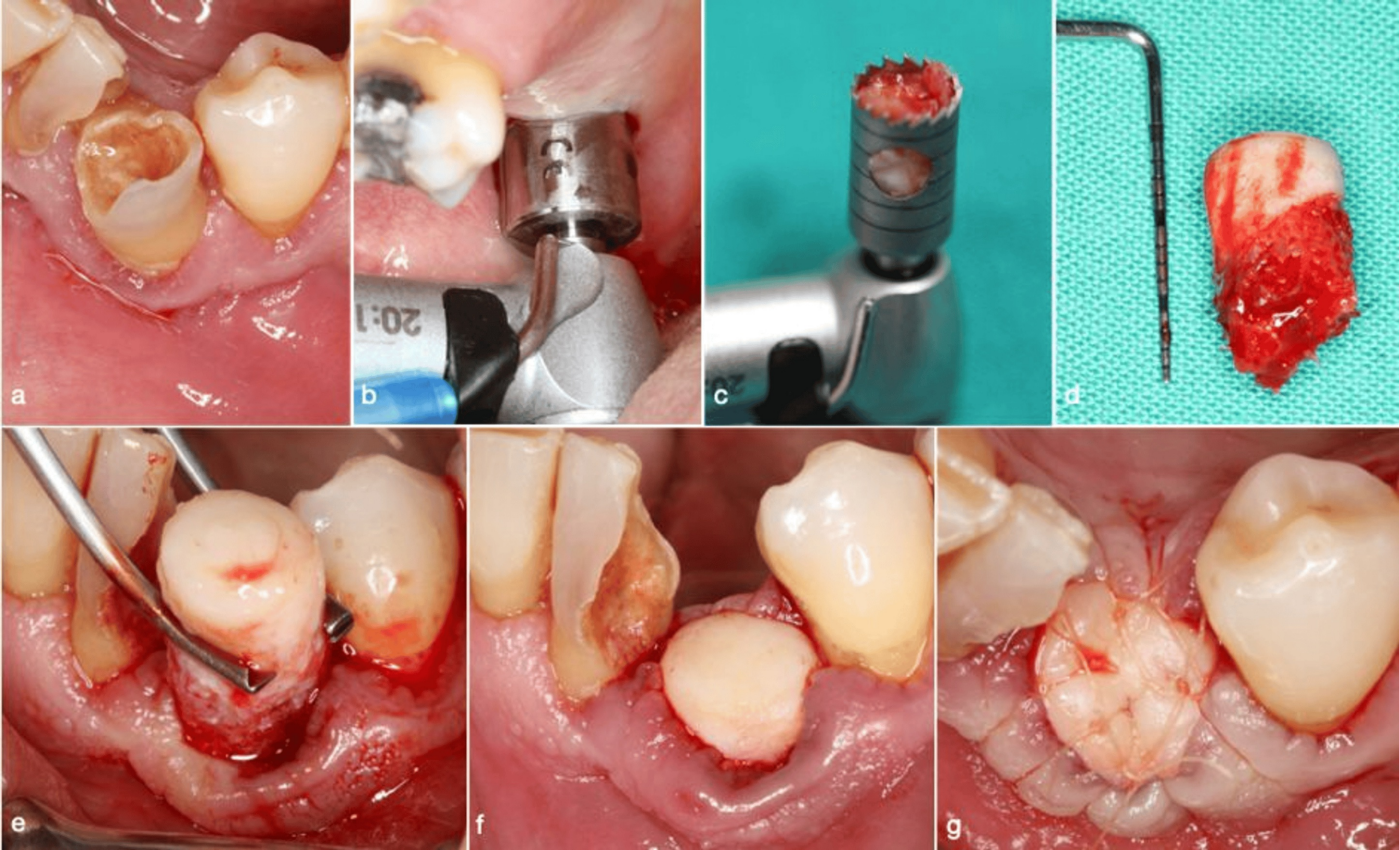
A series of clinical images illustrating the one-piece autologous tuberosity graft technique. (a) Preoperative condition of tooth #43, indicated for extraction due to severe buccal bone deficiency and mobility; (b) a tissue punch is being utilized at the donor site in the right maxillary tuberosity; (c) a bone trephine bur containing the harvested one-piece graft; (d) the extracted one-piece graft; (e) adaptation of the graft within the extraction socket; (f) precise placement of the graft into the socket; and (g) suturing was performed to secure and stabilize the graft.

For autograft bone, a tuberosity block and a greater palatine nerve block were administered. A tissue punch (Meisinger) and trephine bur were used to extract a gingivo-osseous graft. The graft was sculpted, placed in the socket, and secured with 6/0 absorbable sutures. Any excess soft tissue was removed (Figure 3).

All patients were prescribed amoxicillin with clavulanic acid (Augmentin; GlaxoSmithKline, Brentford, UK) at a dose of 1 g twice daily (BID) for seven days, starting on the day of extraction. Ibuprofen (Brufen; Abbott Laboratories, Chicago, IL) 400 mg was recommended three times daily (TID) for pain management. A 0.12% chlorhexidine mouthwash was prescribed TID for 21 days, starting the day after extraction.

Radiographic analysis procedure

For each patient, high-resolution preoperative CBCT scans (T0) were obtained to assess the baseline dimensions of the buccal wall, alongside post-operative CBCT scans (T1) captured approximately 5 to 6 months following socket preservation to evaluate the treatment outcomes.

**Figure 4:**
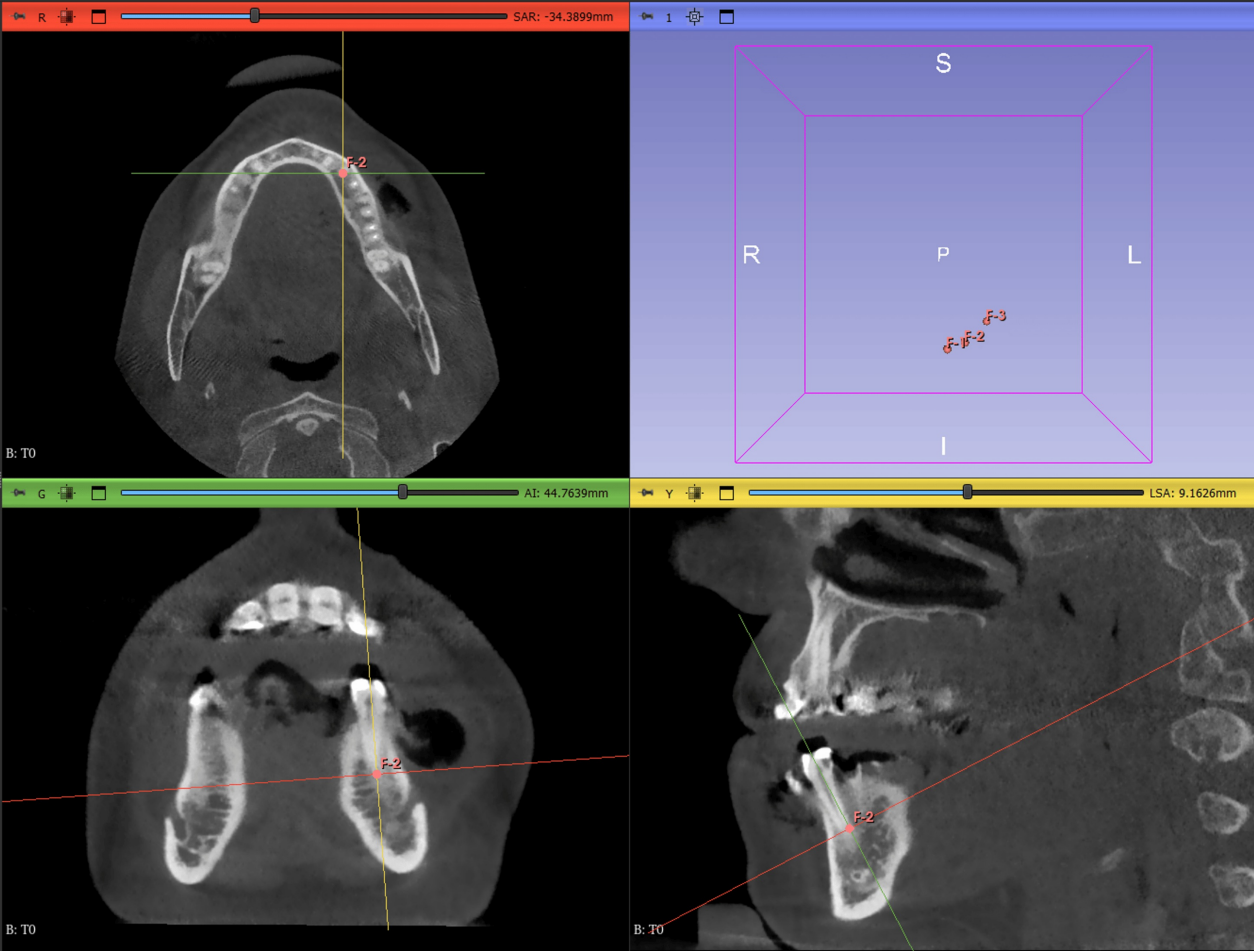
Image showing, in 3D Slicer software, the selection of the same three reference points on T0 and T1 for accurate alignment.

The measurements were performed using 3D Slicer software, which facilitated the superimposition of pre- and post-operative DICOM files. The superimposition process involved selecting three reference points on the pre-operative CBCT, typically at the apex of the teeth, to minimize distortions if it was selected on the crowns. These same three points were then selected on the corresponding post-operative CBCT scan to ensure accurate alignment (Figure 4). 

To enhance the accuracy of buccal wall measurements following superimposition, a multi-software approach was employed, integrating Blue Sky Bio (Blue Sky Bio, LLC, Grayslake, IL), Medit Design (Medit Corp., Seoul, South Korea), and 3D Slicer (Slicer Community, Boston, MA). The initial challenge was the inadequate visualization of the buccal bone in the superimposed models, which made direct measurement difficult. To overcome this, a structured workflow was developed. The process began with AI-based segmentation in Blue Sky Bio, where the jaws were isolated from CBCT scans at two time points: T0 (preoperative scan) and T1 (postoperative scan). This segmentation allowed for the creation of separate 3D models for each jaw. Next, the segmented models were imported into Medit Design in *STL (Standard Tessellation Language) file format* for precise alignment with the T1-on-T0 superimposed model exported from 3D Slicer in *STL file format*. The alignment was performed in two steps: first, aligning the segmented T0 jaw with the T1-on-T0 model, and then aligning the segmented T1 jaw with the same reference model. This process generated two newly aligned models, T0-1 and T1-1, ensuring consistent spatial positioning. These refined models in “STL file format” were then imported back into 3D Slicer, where they were overlaid with the T1-on-T0 model. The combination of segmentation and precise alignment allowed 3D Slicer to automatically delineate anatomical structures, including the buccal wall. With the buccal wall now clearly visualized in both T0 and T1, accurate linear measurements could be performed, significantly enhancing the precision and reliability of the analysis.

**Figure 5:**
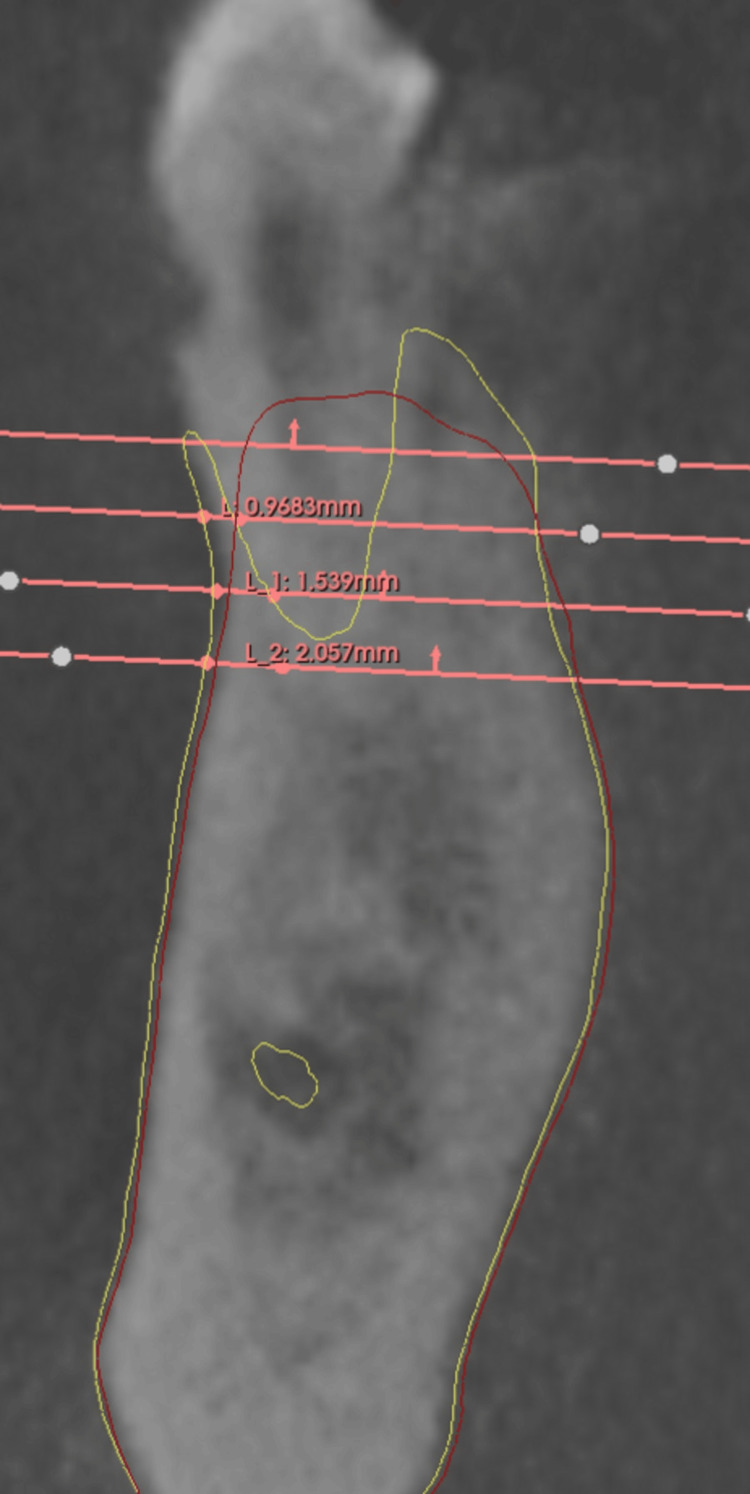
Image showing on 3D Slicer the drawn buccal wall on T0 (yellow representing the initial buccal wall), and linear measurements taken on T0.

Four distinct levels were identified: L0, L2, L4, and L6. Level L0, near the crestal bone, served as a reference due to its susceptibility to remodeling but was not used for quantitative measurements. Instead, it standardized the positioning of deeper measurement levels (L2, L4, L6), spaced 2 mm apart. The exclusion of quantitative measurements at Level L0 (crestal level), which was instead used solely as a visual reference point to standardize the positioning of deeper levels (L2, L4, L6). This decision was based on the high variability and instability of the crestal bone area in the early healing phase, as it is particularly susceptible to remodeling, mechanical trauma during extraction, and postoperative soft tissue changes. Measuring at L0 would have introduced inconsistencies due to its unpredictable remodeling behavior, which often occurs independently of graft material. Additionally, statistical modeling showed that including L0 would not significantly alter outcome comparisons, as meaningful trends were already evident from L2 onwards. Thus, excluding L0 allowed for more reliable, reproducible, and biologically relevant assessments of buccal bone changes while maintaining internal consistency across all cases. This structured multi-software workflow ensured that linear measurements performed at each level in 3D Slicer were highly reproducible. A reference line was created on the axial plane, anchored at the anatomical limit of the tooth on T0, with the second point extending horizontally to the outermost buccal wall boundary (Figure 5). Upon shifting to the post-operative CBCT scan (T1), the reference points and planes remained stable due to the superimposition process, ensuring that all anatomical landmarks from T0 were precisely aligned.

**Figure 6:**
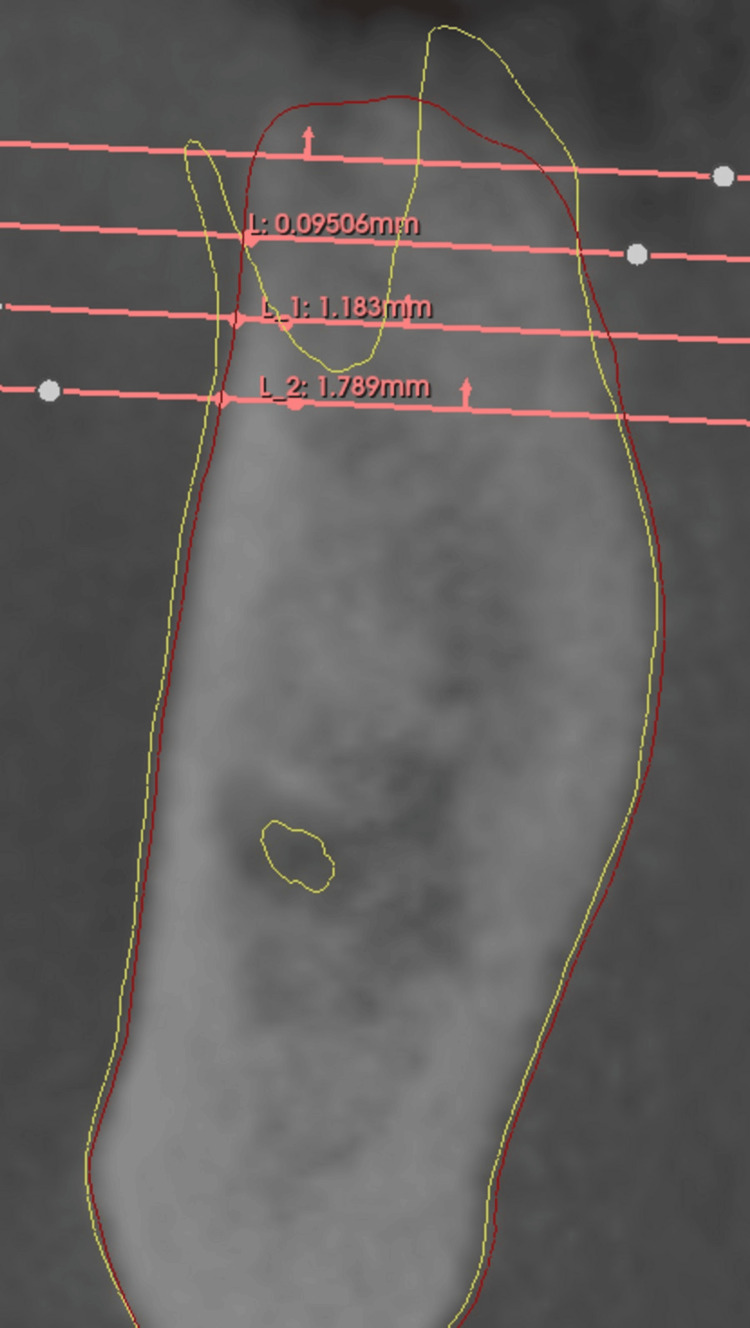
Image showing, in 3D Slicer, the drawn buccal wall on T1 (red representing the final buccal wall) and the linear measurements taken on T1.

As a result, the points marking the anatomical limit of the tooth remained fixed in their original positions, preserving the accuracy of the reference framework. At each measurement level (L2, L4, and L6), the second point of the reference line, which was initially positioned at the outer boundary of the buccal wall on T0, was dynamically adjusted on the axial plane to align with the new buccal wall limit observed on T1 (Figure 6). This allowed for the direct quantification of changes in buccal wall thickness over time.

Measurements were then recorded in an Excel sheet, and the resorption rate (%) was calculated using the following formula, ready to be sent for statistical analysis.

Resorption Rate (%) = ((T0-T1)/T0) ×100

**Figure 7:**
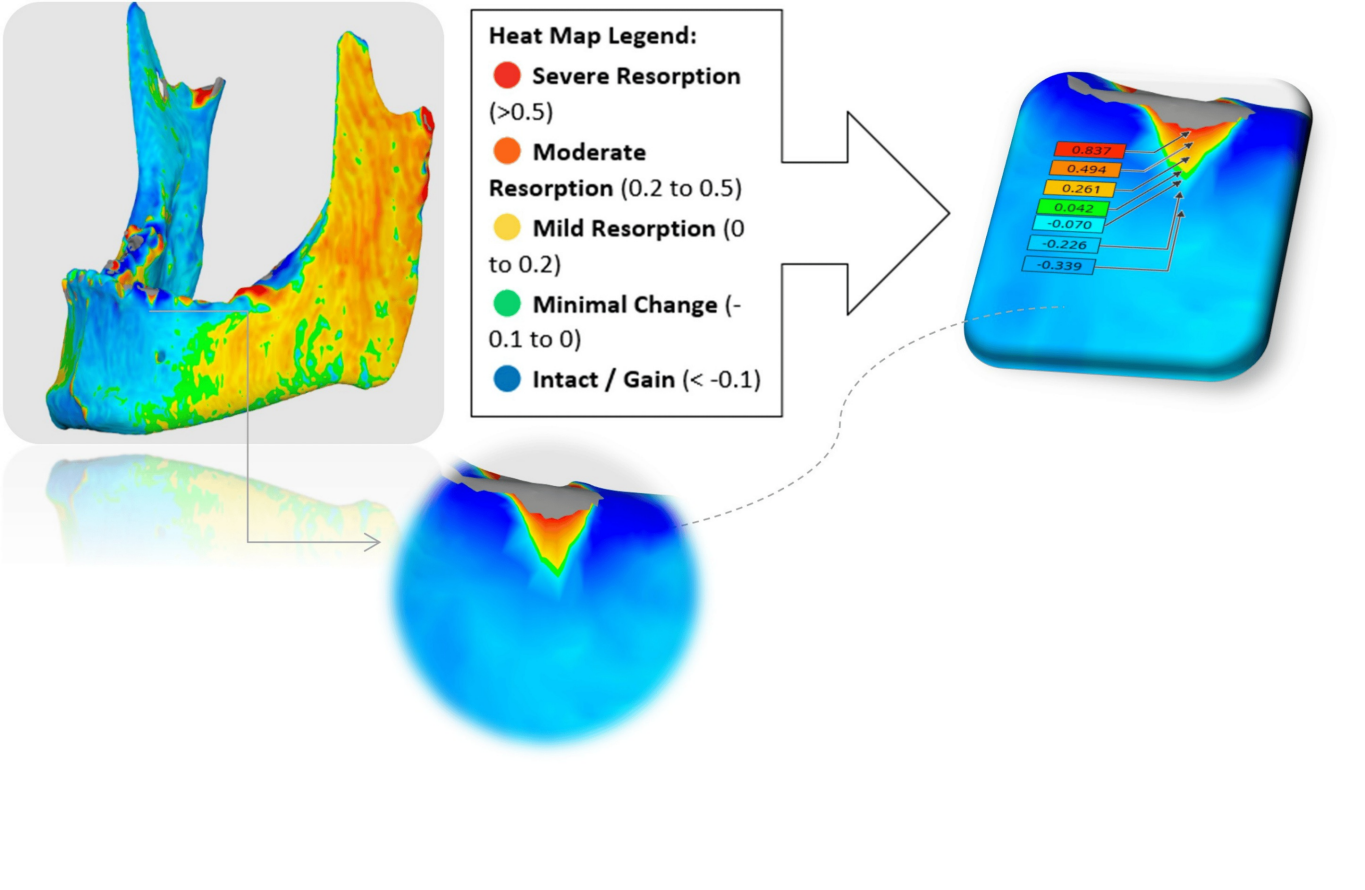
A heat map displaying color-coded bone resorption, ranging from red (high resorption) to blue (intact).

On Medit design, rigid alignment and superimposition of T1 onto T0 (models exported from Blue Sky as STL file format) created a composite image for assessing buccal wall changes. A heat map was generated to visualize bone gain (apposition) or loss (resorption), allowing for a comparative analysis of the three bone substitute materials used in socket preservation (Figure 7).

Statistical analysis

To compare age across different bone substitutes (allografts, xenografts, and autografts), a Kruskal-Wallis test or analysis of variance (ANOVA) was employed, depending on the normality of the data (assessed by the Shapiro-Wilk test).

To compare gender groups across different bone substitutes (allografts, xenografts, and autografts), a chi-square test was employed.

To assess the impact of different bone substitutes (allografts, xenografts, and autografts) on buccal wall dimensions in socket preservation procedures at T0 and T1, a Kruskal-Wallis test or ANOVA was employed depending on the normality of the data (assessed by the Shapiro-Wilk test).

To assess the impact of different bone substitutes (allografts, xenografts, and autografts) on buccal wall resorption rate in socket preservation procedures, a Kruskal-Wallis test or ANOVA was employed depending on the normality of the data (assessed by the Shapiro-Wilk test).

To compare buccal wall dimensions between T0 and T1 at different levels (L2, L4, and L6), a paired t-test or Wilcoxon signed-rank test was employed depending on the normality of the data (assessed by the Shapiro-Wilk test).

Statistical analysis was performed using SPSS software (IBM Corp., New York, USA; version 2017). The significance level was set at 0.05.

## Results

Demographics

Age 

The study population consisted of 23 patients, of whom 8 were randomly assigned to the Allograft group, 7 to the Autograft group, and 8 to the Xenograft group. There was no statistically significant difference in age between the groups (54.4 ± 6.9 vs. 46.6 ± 6.8 vs. 55.8 ± 11.4 years, respectively; *P* > 0.05).

Gender

There was also no statistically significant difference in gender distribution between the groups (number of females: 3 vs. 6 vs. 4, respectively, for the allograft, autograft, and xenograft groups; *P* > 0.05).

Buccal wall dimensions across different bone substitutes

 *At T1*

The Kruskal-Wallis test revealed that Xenografts were associated with bigger dimensions than autografts and allografts at level L2 (*P* = 0.02 and *P* = 0.04, respectively).

**Figure 8:**
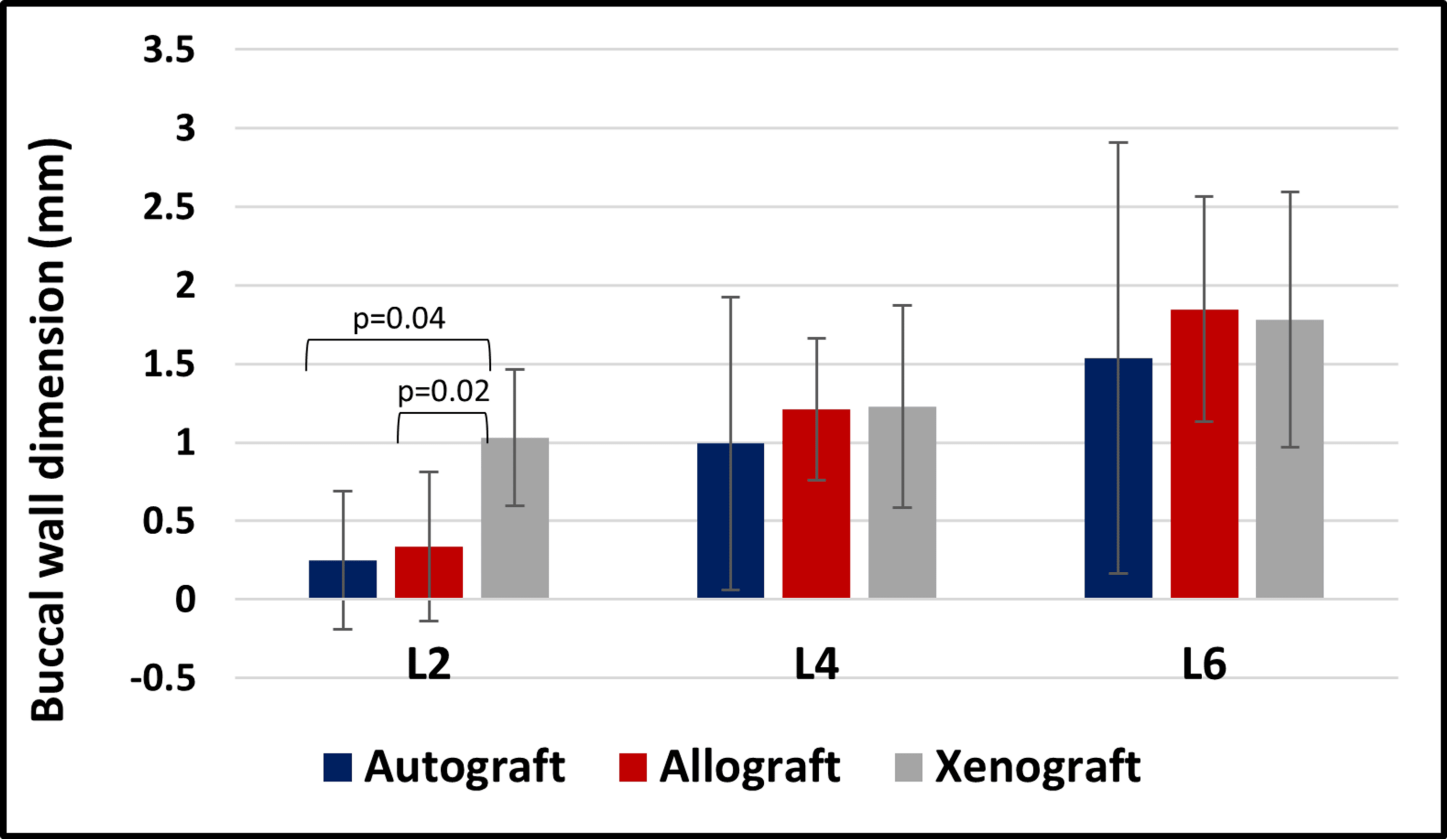
Graphical representation of the buccal wall dimension variations across different bone substitutes at T1.

The Kruskal-Wallis test did not reveal any statistically significant differences in buccal wall dimensions across different bone substitutes at levels L4 and L6 (*P *> 0.05; Figure 8).

Resorption rate across different bone substitutes

**Figure 9:**
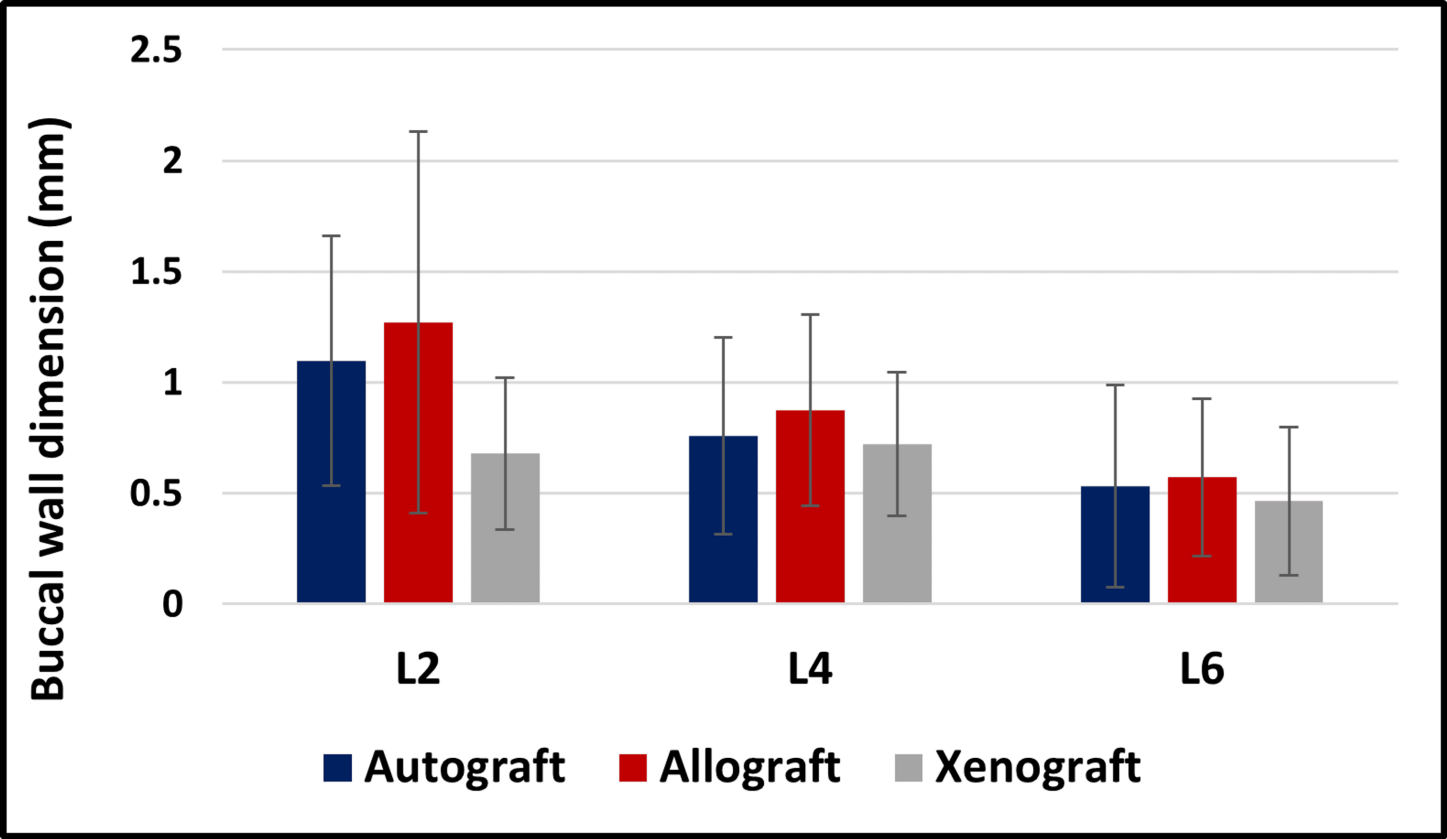
Graphical representation of resorption rates of the buccal wall across different bone substitutes.

The Kruskal-Wallis test did not reveal any statistically significant differences in resorption rate across different bone substitutes at levels L2, L4, and L6 (*P *> 0.05; Figure 9).

Buccal wall dimensions across time points

**Figure 10:**
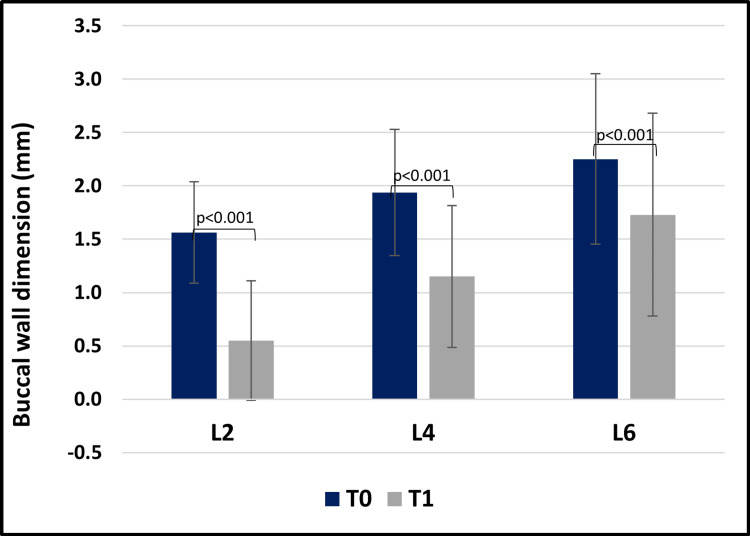
Graphical representation of buccal wall dimensions at different levels across time points (T0 vs. T1).

The Wilcoxon signed-rank test revealed that buccal wall dimensions had higher values at T0 compared to T1, at L2, L4, and L6 (*P *< 0.001; Figure 10).

## Discussion

This study provides valuable insights into the role of three commonly used bone substitutes: allografts, xenografts, and autografts, in socket preservation procedures, with particular focus on their influence on buccal wall dimensional changes. Socket preservation remains a cornerstone in implantology and restorative dentistry, especially in esthetically demanding regions where ridge contour and stability are critical for long-term functional and cosmetic outcomes. Within this context, understanding the comparative performance of grafting materials is essential for evidence-based clinical decision-making.

Our demographic analysis demonstrated that the distribution of age and gender across the three groups was statistically similar, thereby ensuring that any differences observed in outcomes could be confidently attributed to the properties of the grafting materials themselves rather than patient-related factors. Establishing this homogeneity at baseline was important for strengthening the internal validity of the study.

At baseline (T0), buccal wall dimensions were comparable across all groups (*P *> 0.05), providing a uniform starting point for subsequent evaluation. At the follow-up (T1, 5-6 months), significant differences were observed at the coronal level (L2), where xenografts preserved buccal wall thickness more effectively than autografts and allografts (*P *= 0.02 and *P* = 0.04). This finding suggests that xenografts provide superior support in the early stages of healing, which is crucial for maintaining ridge contour in esthetically sensitive regions such as the anterior maxilla. However, at the mid-root (L4) and apical (L6) levels, no significant differences were observed among the three materials (*P* > 0.05), implying that all graft types perform comparably in deeper areas.

While xenografts demonstrated a superior ability to preserve coronal thickness at L2, the overall rate of buccal wall resorption did not differ significantly among the groups (*P* > 0.05). This indicates that although xenografts initially preserve ridge contour more effectively, their resorption trajectory over time resembles that of autografts and allografts. The absence of statistical significance could be linked to the study’s relatively small sample size (*n *= 23), since larger cohorts are required to detect more subtle differences. The resorption chart (Figure 9) nonetheless highlighted observable trends: xenografts exhibited the least resorption, autografts intermediate levels, and allografts the most pronounced resorption across all measured levels.

**Figure 11:**
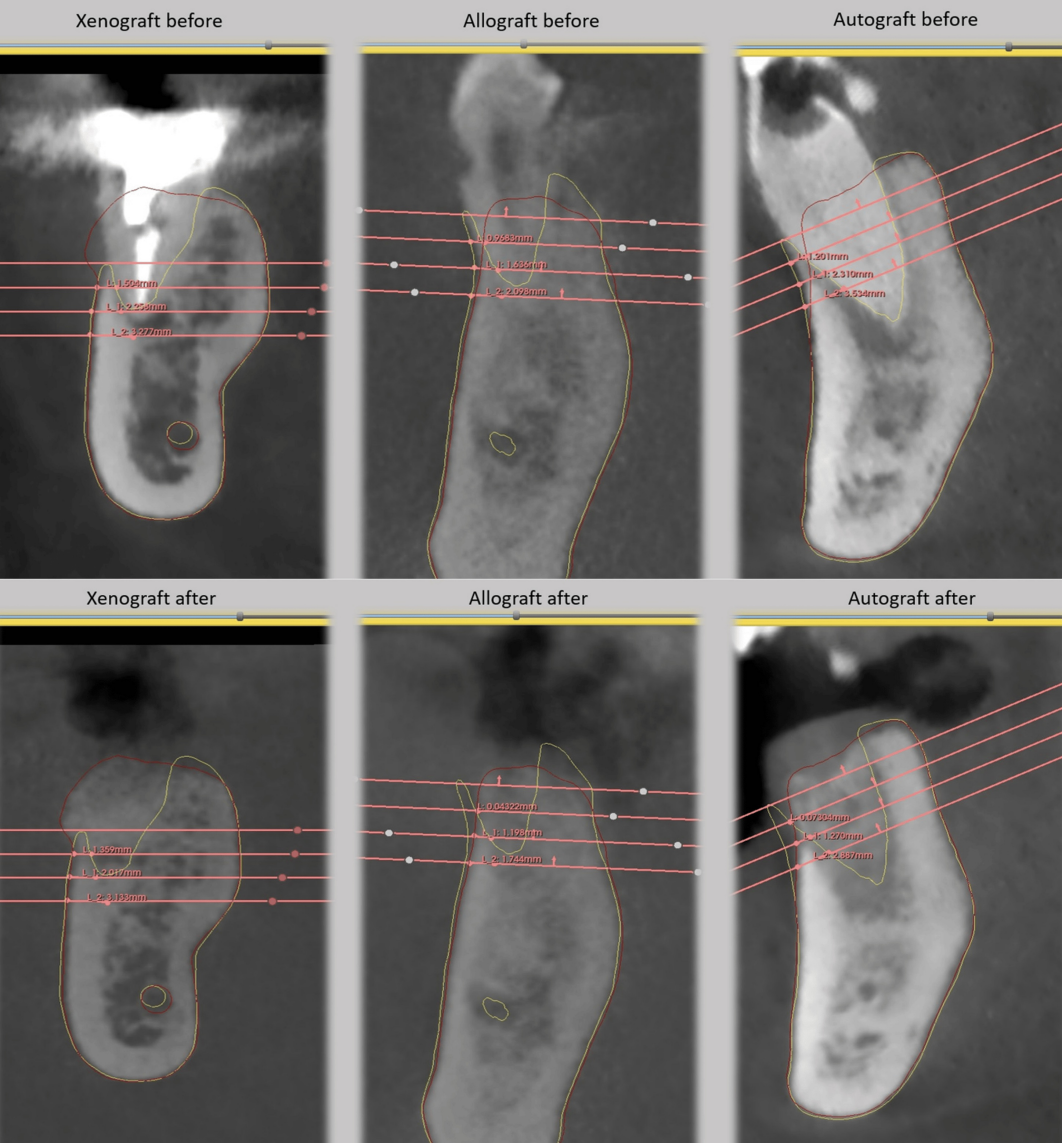
Comparison of linear CBCT measurements of buccal wall dimensions before and after grafting with xenograft, allograft, and autograft bone substitutes. CBCT, cone beam computed tomography

Qualitative observations supported these quantitative findings. The xenograft (Bio-Oss) group, which initially presented with thin concave buccal walls, displayed clear contour preservation and even gain post-grafting. The buccal wall appeared thicker, more continuous, and better integrated; findings confirmed by linear measurements. The allograft group, which also began with compromised buccal walls, showed significant improvement after grafting, though not as pronounced as the xenograft group. In contrast, the autograft group demonstrated the least favorable changes, with limited increases in buccal wall thickness and less effective contour preservation. Linear measurement analysis thus consistently ranked performance as xenograft > allograft > autograft (Figure 11).

**Figure 12:**
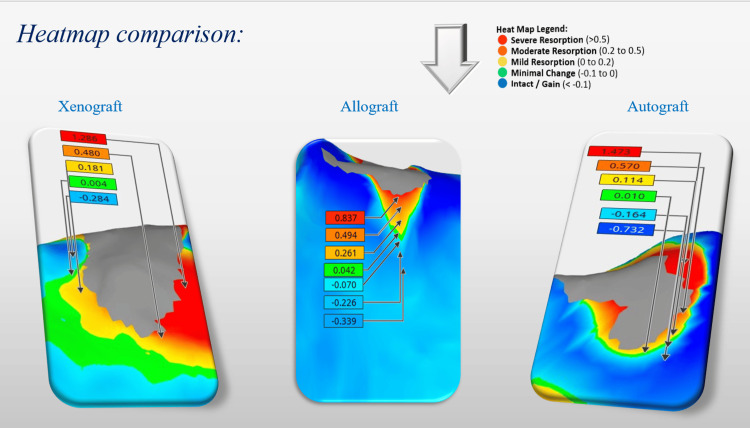
Heatmap comparison of buccal wall resorption following xenograft, allograft, and autograft procedures.

Heatmap analysis further illustrated these differences in a visually striking manner. Xenografts exhibited large regions of minimal change or slight gain (blue/green), and only small areas of moderate resorption (orange/red), reflecting superior dimensional stability. Allografts demonstrated moderate resorption with mixed green, yellow, and some orange areas, indicating a less stable outcome than xenografts but superior to autografts. Autografts, however, showed the greatest degree of resorption, with extensive red and orange zones, and minimal areas of volume gain. These visual representations corroborated the quantitative data, reinforcing xenografts’ superior stability at the coronal level (Figure 12).

Despite the performance differences, the study also confirmed that significant buccal wall resorption occurred in all cases (*P *< 0.001). This finding aligns with the broader literature on post-extraction bone loss, which highlights the persistent challenge of alveolar ridge resorption even when grafting materials are employed. It underscores the importance of additional or adjunctive techniques such as guided bone regeneration (GBR), soft tissue augmentation, or strategic implant timing (delayed placement) when ridge preservation is a clinical priority [22,23,24].

The current findings resonate with previous literature. For example, Binkhorst et al. [25] and Apaza-Bedoya et al. [26] also demonstrated xenografts’ superiority in preserving coronal buccal wall thickness and maintaining ridge contour in esthetic areas. These parallels strengthen the argument that xenografts are particularly advantageous in the anterior maxilla, where cosmetic demands are highest. Conversely, our results diverge from those of Čandrlíć et al. [20], who reported slower xenograft resorption compared with autografts and allografts. In our study, resorption patterns were broadly similar across materials, likely due to the shorter follow-up period and smaller sample size, which may have limited the ability to capture subtle long-term differences.

Supporting this interpretation, Avila-Ortiz et al. [27] similarly observed convergent resorption patterns across graft types when follow-up duration was limited.

Findings from Ten Heggeler et al. [28] further align with ours by reporting no significant differences in preservation at deeper levels, underscoring that xenografts’ advantage is largely restricted to the coronal dimension. This suggests that while graft materials can mitigate initial resorption, long-term outcomes at deeper sites become comparable.

Regarding autografts, our results contrast with Khairallah et al. [29], who emphasized their osteogenic potential and reported superior bone regeneration. While autografts are often regarded as the “gold standard” due to their osteogenic and osteoconductive properties, in the context of socket preservation, they did not outperform xenografts or allografts in this study. This could be explained by variations in harvesting technique, graft form (block vs. particulate), or the healing dynamics of extraction sockets compared to larger reconstructive sites.

The literature also suggests xenografts provide extended structural support during remodeling, as highlighted by Cardaropoli et al. [30], who observed slower resorption rates in long-term follow-up. Our shorter observation period may not have been sufficient to capture this characteristic, highlighting the need for extended follow-up studies to fully assess long-term outcomes.

In light of these findings, the null hypothesis (H0) is partially rejected. Graft materials clearly influence buccal wall preservation in a site-dependent manner: xenografts demonstrate superior outcomes at the coronal level, while deeper levels show no significant differences among materials. This emphasizes that although xenografts may provide early esthetic benefits, the overall resorption process appears to converge across graft types over time.

One of the strengths of this study lies in its methodological rigor. The use of CBCT imaging, 3D superimposition, and AI-based segmentation provided highly precise and reproducible measurements of buccal wall changes. This advanced workflow minimized alignment errors and allowed for both anatomical and clinical evaluation of graft performance. Furthermore, the comparative design involving three graft types enhanced the clinical applicability of the findings by directly addressing material selection in socket preservation. Importantly, this research represents the first study of its kind conducted in Lebanon, adding valuable regional data to the global discourse on bone grafting.

Nevertheless, several limitations must be acknowledged. The small sample size (*n* = 23), distributed unevenly among groups, reduced the statistical power and increased the risk of type II error. The relatively short follow-up (5-6 months) provided useful insights into early remodeling but may not fully reflect the long-term resorption trajectory, particularly of xenografts. Moreover, while CBCT and AI-based analysis provided accurate radiographic data, the absence of histologic and soft-tissue evaluation limited the ability to assess biological integration and soft-tissue response. Additionally, heterogeneity in surgical approaches and graft application methods may have influenced results. For example, allografts were combined with d-PTFE membranes, xenografts with collagen tape, and autografts were harvested as block grafts, each introducing unique healing dynamics. Variability in graft form, powder vs. block, may also have contributed to differences in integration and resorption. Anatomical site variability (anterior vs. posterior, maxilla vs. mandible) further complicated interpretation, as regional differences in bone density, vascularization, and mechanical load could influence healing outcomes. Another limitation of this study is the absence of a non-grafted control group. Including such a group would have enabled direct comparison between spontaneous post-extraction remodeling and graft-mediated buccal wall preservation, allowing clearer quantification of the true benefit of each graft material. However, the omission of a control group was ethically justified, as socket preservation is widely considered standard care in sites intended for future implant placement. Withholding grafting in these cases could have compromised ridge dimensions and adversely affected implant outcomes.

Future research should employ larger sample sizes, longer follow-up durations, and standardized protocols to minimize confounding factors. Controlled studies in non-implant or delayed-treatment sites, together with histological and soft tissue assessments, would help clarify the extent to which grafting modifies natural resorption patterns. Additionally, site-specific subgroup analyses may further elucidate differences in grafting outcomes between anterior and posterior regions, as well as between maxillary and mandibular sites.

## Conclusions

In conclusion, the findings of this study indicate that socket preservation plays a crucial role in maintaining alveolar ridge dimensions, particularly in the buccal wall, which is highly prone to resorption. Among the grafting materials evaluated, allografts, xenografts, and autografts were effective in minimizing bone loss; however, xenografts demonstrated superior preservation at the coronal level (L2), making them particularly advantageous in esthetic areas. At deeper levels (L4 and L6), no significant differences were observed among the materials, suggesting that long-term resorption patterns may be comparable. Although the differences in buccal wall resorption were not statistically significant, xenografts showed the least resorption trend, followed by autografts and allografts, consistent with previous findings highlighting the prolonged structural stability of xenografts. Despite these favorable outcomes, notable buccal wall resorption was observed in all groups, underscoring the possible need for adjunctive techniques such as guided bone regeneration or soft tissue augmentation. Further prospective studies with larger sample sizes and longer follow-up periods are recommended to validate these results and gain deeper insights into the long-term dimensional stability of different grafting materials.
